# Molybdenum impregnated g-C_3_N_4_ nanotubes as potentially active photocatalyst for renewable energy applications

**DOI:** 10.1038/s41598-021-96490-6

**Published:** 2021-08-19

**Authors:** Naseer Iqbal, Adeel Afzal, Ibrahim Khan, Muhammad Shahzeb Khan, Ahsanulhaq Qurashi

**Affiliations:** 1 Department of Chemistry, College of Science, University of Hafr Al Batin, P.O. Box 1803, Hafr Al Batin, 39524 Saudi Arabia; 2 Center of Research Excellence in Nanotechnology, King Fahd University of Petroleum and Minerals, Dhahran, 31261 Saudi Arabia; 3 Department of Mechanical Engineering, College of Engineering, University of Hafr Al Batin, P.O. Box 1803, Hafr Al Batin, 39524 Saudi Arabia; 4 Department of Chemistry, Khalifa University of Science and Technology, Main Campus, P.O. Box 127788, Abu Dhabi, United Arab Emirates

**Keywords:** Photocatalysis, Energy

## Abstract

Molybdenum (Mo) impregnated g-C_3_N_4_ (Mo-CN) nanotubes are fabricated via a thermal/hydrothermal process to augment photoelectrochemical properties during solar-driven water-splitting (SDWS) reactions. Graphitic-C_3_N_4_ is an attractive material for photocatalysis because of its suitable band energy, high thermal and chemical stability. The FE-SEM and HR-TEM comprehend the nanotube-like morphology of Mo-CN_._ The spectroscopic characterization revealed bandgap energy of 2.63 eV with high visible-light activity. The x-ray diffraction of pristine g-C_3_N_4_ and Mo-CN nanotubes discloses the formation of triazine-based nanocrystalline g-C_3_N_4_, which remains stable during hydrothermal impregnation of Mo. Furthermore, Mo-CN nanotubes possess high sp^2^-hybridized nitrogen content, and metallic/oxidized Mo nanoparticles (in a ratio of 1:2) are impregnated into g-C_3_N_4_. The XPS analysis confirms C, N, and Mo for known atomic and oxidation states in Mo-CN. Furthermore, high photocurrent efficiency (~ 5.5 mA/cm^2^) is observed from 5%-Mo-CN nanotubes. That displays efficient SDWS by 5%-Mo-CN nanotubes than other counterparts. Impedance spectroscopy illustrated the lowest charge transfer resistance (R_*ct*_) of 5%-Mo-CN nanotubes, which further confirms the fast electron transfer kinetics and efficient charge separation resulting in high photocurrent generation. Hence, 5%Mo-CN composite nanotubes can serve as a potential photocatalytic material for viable solar-driven water splitting.

## Introduction

The environmental and energy crises are foreseeable major problems of the impending decades for living beings due to overexploitation of natural resources^1,2^. Among these, non-renewable energy sources such as fossil fuels have been extensively used for fulfilling day-to-day energy requirements. That not only slackens the available resources but also accounts for many health and environmental concerns. The researchers are working hard to combat these issues and ascertain new solutions. Hence, the concept of clean and green alternative energy resources^3^ came into existence. Among various alternative energy processes, the concept of solar-driven water splitting to produce hydrogen as a clean and green fuel is grabbing the utmost attention^4,5^. Since the first report on photocatalytic reduction of water into hydrogen and oxygen^6^, tremendous research efforts have been focused on developing efficient photocatalysts to achieve solar-driven water splitting for more economical alternative energy resources ^7–10^.

Metal supported and metal-free photocatalysts have been widely explored for water splitting reactions^9–13^. Besides, there is a great deal of scientific interest in metal-free polymeric graphitic carbon nitride (g-C_3_N_4_) because of its suitable physicochemical properties such as chemical and thermal stability, unique band structure, and its utilization in photocatalytic degradation of organic pollutants, photocatalytic CO_2_ reduction/conversion, and hydrogen production by solar-driven water splitting^11,12,14,15^. However, the photocatalytic activity of g-C_3_N_4_ still suffers from low conversion efficiencies due to the rapid electron–hole recombination or simultaneous charge recombination, low electrical conductivity, low optical absorption, and small surface area^16^. Therefore, fabrication of nanostructured and-or mesoporous g-C_3_N_4_ materials^17^ and doping with suitable metals or non-metals^1,18–20^ are proposed to address these problems^21^.

Impregnation or doping metals into g-C_3_N_4_ nanostructures are amongst the most suitable strategies to enhance the optical and photoelectrochemical properties of g-C_3_N_4_ through the fabrication of innovative heterojunction nanostructures. Furthermore, metal doping can modify the electronic structure of semiconductors and their textural properties, thus improving their photocatalytic activity^21,22^. For instance, the photoelectrochemical performance of g-C_3_N_4_ nanomaterials is significantly improved by Fe and Ti doping^11,23–25^. These studies revealed increased surface area, narrower bandgap, well-aligned band structure, and photodegradation because of the enhanced optical absorption and faster rate of charge carrier transfer^11,23–25^. On the other hand, bearing some outstanding characteristics mentioned above, such g-C_3_N_4_ photocatalysts showed poor carrier properties, short hole diffusion length, excitation span, weak career mobility, and shallow light penetration depth resulting in decreased water oxidation on exposure to visible light^26,27^. These issues can be addressed by tuning morphology by surface modifications, reducing bang gap energy and overpotentials for enhanced photocurrent density during SDWS. In SDWS, reduced bang gap is important, as the redox reaction occurs in the electronic structure of the photocatalyst, i.e., illustrated by a conduction band (CB) and a valence band (VB) discerned by bandgap energy (E_g_). Under visible light, the photocatalyst is excited by photons with energy equal to or greater than the bandgap energy (hv ≥ E_g_). Thus, electrons receiving higher energy from the photons are pushed from VB to CB. In turn, the electrons and holes are transferred to the surface of the photocatalyst undergo reduction or oxidation^28^. These characteristics can be achieved either by growing nanosheets and nanotubes or by incorporating metals and corresponding cocatalysts into the host materials or both. As a result, one can alter electronic band structures for better photocatalytic performance and improved electron and hole transport^26^.

Considering the exceptional electrical, optical, and catalytic properties of Molybdenum (Mo) among transition metals for advanced energy applications, comprehensive studies investigate its photocatalytic reccital^29–34^. Mo-based materials exhibited excellent electrical conductivity, enhanced charge carrier mobility, and a variable(~ 1.2–2.2 eV) bandgap energy^21,35–38^. Guo et al.^39^ reported that Mo in the form of Mo(IV) when incorporated into the host materials, not only introduces localized electron-trapping states at the bottom of the conduction band but also elevates the Fermi level towards the defect level, which endows the doped system with enhanced n-type characteristic and the defect state with strong electron-trapping ability. Moreover, a nonuniform distribution of charge density is formed for the Mo-doped materials, facilitating the separation of photoexcited charge carriers. Therefore, the Mo-doped materials exhibit remarkably enhanced photocatalytic activity, making Mo a suitable material to enhance the photocatalytic water splitting nature of g-C_3_N_4_. Different strategies are known to prepare 1-D or 2-D Mo-based nanomaterials, such as mechanical exfoliation^40^, chemical exfoliation^41^, chemical vapor deposition (CVD)^42^ hydro, and solvothermal^30,43,44^ methods, etc.

Herein, we present a straightforward approach to prepare Molybdenum (Mo) impregnated g-C_3_N_4_ nanotubes via a thermal and hydrothermal route. The surface morphology and elemental composition are characterized via FE-SEM, HR-TEM, XRD, and XPS. Which analyses reveal the nanotubes-like morphology of molybdenum-doped g-C_3_N_4_ (Mo-CN), high purity, and crystalline structure. Furthermore, high nitrogen content and reduced bandgap prove decisive factors for improved optical and photoelectrochemical properties of Mo-CN nanotubes. Photoelectrochemical (PEC) measurements of Mo-CN nanotubes exhibit good photocurrent generation with excellent stability under 1 Sun solar irradiation source, low charge transfer resistance (R_*ct*_), fast electron transfer kinetics, and efficient charge separation. Thus, it supports the hydrothermally fabricated Mo-CN nanotubes with great potential for efficient solar-driven water splitting.

## Materials and methods

### Materials

High purity, analytical grade chemicals, solvents, and reagents were obtained and used as received without further purifications unless otherwise indicated. Ammonium heptamolybdate tetrahydrate, melamine, ethanol, and acetone were purchased from Millipore Sigma and were used as received. Deionized water was used for all experiments and solutions, including solutions used in photoelectrochemical (PEC) measurements.

### Synthesis of molybdenum-impregnated graphitic carbon nitride (Mo-CN) nanotubes

**Figure 1:**
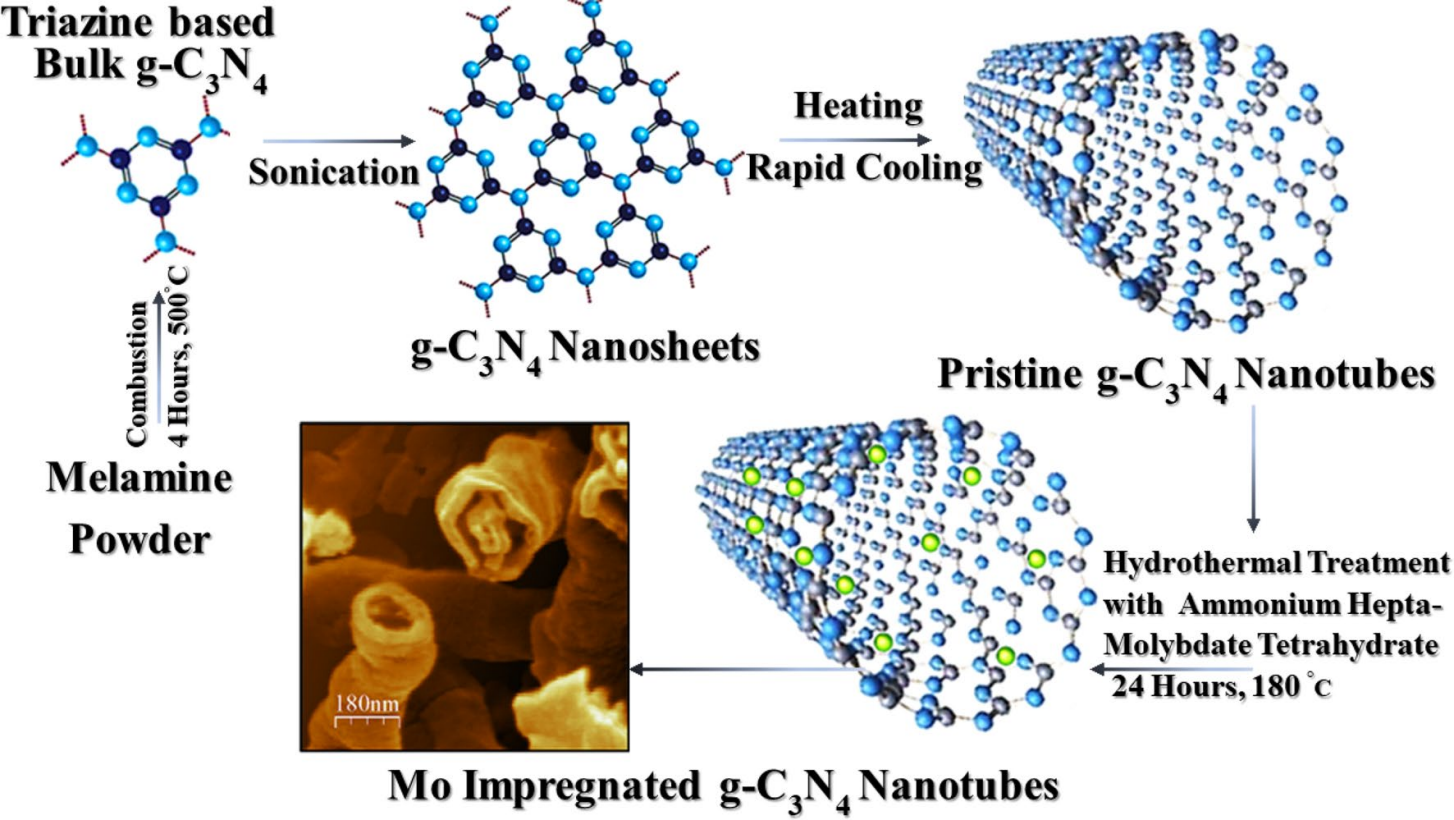
Schematic synthesis of Molybdenum impregnated graphitic carbon nitride nanotubes: Pristine g-C_3_N_4_ nanotubes are prepared in the first step through the combustion of melamine at 500^◦^C following sonication of bulk g-C_3_N_4_ to obtain bulk g-C_3_N_4_ nanosheets followed by and heating-rapid cooling of g nanosheets. Later on, Mo-C_3_N_4_ nanotubes are produced by a hydrothermal reaction of ammonium heptamolybdate tetrahydrate at 180 °C. The inset also shows an FE-SEM image of a typical Mo-g-C_3_N_4_ nanotubes sample.

The synthesis of Molybdenum impregnated carbon nitride (Mo- g-C_3_N_4_) nanotubes is carried out as follows. It includes the preparation of bulk graphitic carbon nitride (g-C_3_N_4_) from melamine. First, melamine was annealed in the air for 4 h at 500 °C in a furnace until a yellowish powder (g-C_3_N_4_) is obtained. Next, the bulk g-C_3_N_4_ was exfoliated to prepare g-C_3_N_4_ nanosheets by sonication for 2 h in 70% ethanol. A Morphological transformation strategy was followed with slight modification^45^. Afterward, the as-synthesized g-C_3_N_4_ nanosheets powder was slowly heated at the rate of 10 °C/min up to 300 °C and held at this temperature for 60 min, and then it was transferred into an ice-water bath. Next, the as-prepared g-C_3_N_4_ nanotubes were collected by filtration and vacuum dried at 120 °C for 4 h. In the next step, Molybdenum was impregnated into g-C_3_N_4_ nanotubes by the hydrothermal approach. Several studies support the metals doping into g-C_3_N_4_ via thermal/hydrothermal treatments^39,46–48^. For a hydrothermal reaction, 5% and 15% aliquots (w/w ratio) of ammonium heptamolybdate tetrahydrate and as prepared g-C_3_N_4_ nanotubes powder were mixed in water, respectively. Next, each reaction mixture was sonicated at 60 °C for 1 h. Subsequently, the reaction mixtures were transferred into stainless-steel autoclaves containing Teflon vessels. The hydrothermal reaction proceeded for 24 h at 180 °C. Then, each reaction mixture was centrifuged at 4000 rpm for 5 min. Finally, the products 5% Mo- g-C_3_N_4_ and 15% Mo- g-C_3_N_4_ were collected, washed with deionized water thrice before drying in a vacuum oven for 2 h at 150 °C. Figure 1 shows a schematic of the formation of Mo impregnated g-C_3_N_4_ nanotubes.

### Characterization

X-ray diffraction (XRD) was used to characterize Mo-CN nanotubes via a benchtop MiniFlex X-ray diffraction (mini-XRD) instrument from Rigaku with Cu Kα radiation at 40 kV and 15 mA. XRD patterns were recorded in the range of 10–70° (2θ) at a scanning rate of 3° min^-1^. The structural composition and crystalline phases of Mo-CN nanotubes were determined from the XRD library database. Also, the structural composition and elemental speciation of Mo-CN nanotubes were verified by x-ray photoelectron spectroscopy (XPS) using PHI 5000 Versa Probe II spectrometer (UlVAC-PHI), employing Al Kα as the incident radiation source. The C1s (E = 284.5 eV) served as the internal standard. The nanotubes-like morphology of Mo-CN was observed under TESCAN Lyra 3 field emission dual beam (electron/focused ion beam) system combined high‐end field‐emission scanning electron microscope (FE-SEM). JEOL JEM-2100F transmission electron microscope was used to acquire high-resolution transmission electron micrographs (HR-TEM) at 200 kV. Optical properties of Mo-CN nanotubes were measured by diffused reflectance spectroscopy on Agilent Cary 5000 high-performance UV–Vis-NIR spectrophotometer containing praying mantis accessory with alignment tools and powder cell sample cups. These materials are also tested for photoluminescence studies carried out with fluorolog-3 Imaging Spectrophotometer at an excitation wavelength of 350 nm and a slit width of 2 nm. In contrast, PL emission spectra were acquired by Spectrofluorimeter (JASCO, FP-8500) and FTIR 6700 Nicolet™ Fourier transform infrared (FTIR) spectrometer recorded the vibrational modes in the materials.

### Fabrication of photoanodes and photoelectrochemistry setup

Mo-CN nanotubes were fabricated on fluorinated tin oxide (FTO) conducting glass substrates to study the photoelectrochemical (PEC) characteristics. First, FTO glass substrates were washed by continuous ultrasonication in acetone (10 min) and water (10 min), respectively. In the next step, slurries of Mo-CN nanotubes in 50% (v/v) ethanol/water and 20 µL Nafion mixture were prepared and drop-casted on pre-treated FTO glass substrates separately. Finally, the FTO/Mo-CN nanotube substrates were heated at 110 °C for 2 h to evaporate the solvents and harden the Mo-CN nanotubes films. PEC measurements were carried out in 0.5 M Na_2_SO_4_ solution (pH = 7) using a three-electrode electrochemical setup consisting of FTO/Mo-CN nanotubes as the working electrodes, a Pt wire as the auxiliary electrode, and a standard Ag/AgCl as the reference electrode. PEC experiments were performed with Metrohm Autolab PGSTAT302N potentiostat. For solar-driven PEC measurements, an Oriel Sol 3A class AAA Solar Simulator-Newport (100 mW.cm^−2^), IEC/JIS/ASTM certified, containing a 450 W Xenon lamp, an Air Mass 1.5G Filter, and a 2 × 2 inch aperture for output beam was used.

## Results and discussion

### FT-IR spectroscopy

FTIR spectra of pristine g-C_3_N_4_, 5%Mo-CN, and 15%Mo-CN nanotubes are presented in Fig. 1S (supplementary information). The formation of triazine-based g-C_3_N_4_ structure is confirmed by the presence of characteristics heterocyclic ν(C–N/C = N) stretching vibrations and ν(N–H) shearing vibrations in the range of 1460–1650 cm^−1^. The characteristic ν(C–N/C = N) bands of the condensed triazine units appear at 1250 and 1325 cm^-1^, which are attributed to the stretching vibrations of C–NH–C (partially condensed) and C–N(–C)–C (fully condensed) units, respectively. Furthermore, a sharp peak around 810 cm^-1^ corresponds to the characteristic breathing mode vibrations of triazine units, which further confirms the formation of the g-C_3_N_4_ structure, as demonstrated in Fig. 1. These peak assignments agree with the pertinent literature^49–52^. Furthermore, the broad transmittance peaks exhibited in the range of 3000–3300 cm^-1^ are attributed to the N–H vibrations, i.e., ν(N–H) stretching. The existence of ν(N–H) band in 5%Mo-CN and 15%Mo-CN samples suggests that g-C_3_N_4_ nanotubes remain protonated during the impregnation of Mo metal nanoparticles, which also substantiates the stability of the triazine-based g-C_3_N_4_ structure. However, significant differences in the FTIR spectra of pristine and Mo-impregnated g-C_3_N_4_ nanotubes can be observed in the peak shifts and intensity changes at 1580, 1650, and 3125 cm^−1^, which correspond to stretching and scissoring vibrations of –NH/–NH_2_ groups^49–52^. These changes in the FTIR spectra of Mo-CN nanotubes are attributed to the deposition of Mo nanoparticles on g-C_3_N_4_ surfaces, which results in strong metal coordination with g-C_3_N_4_ nanotubes. It is envisaged that such interactions enhance the potential photoelectrochemical performance.

### Surface morphology

**Figure 2:**
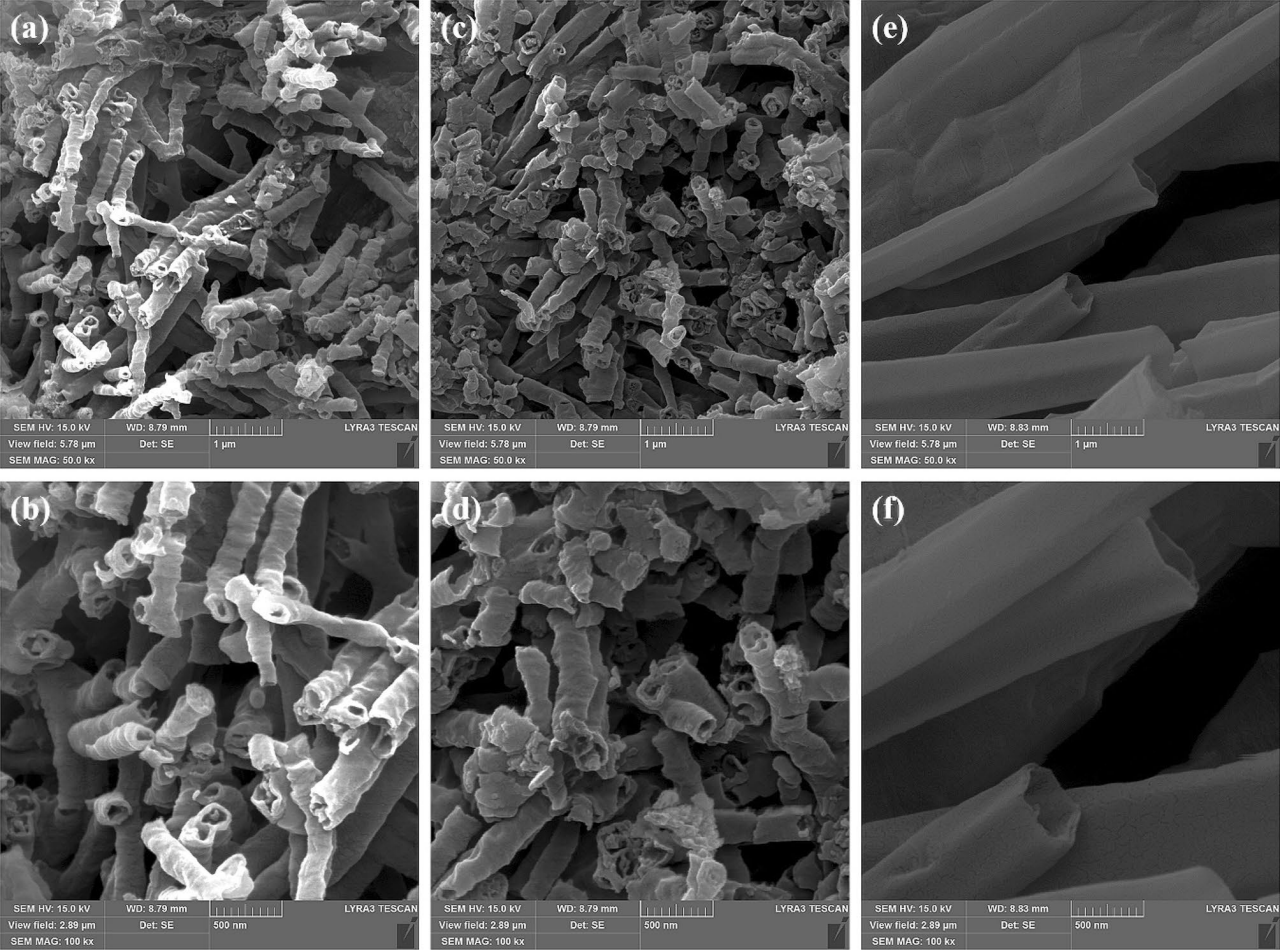
FE-SEM images of the as-prepared, pristine g-C_3_N_4_ and Molybdenum impregnated g-C_3_N_4_ samples: (**a**, **b**) pristine g-C_3_N_4_, (**c**, **d**) 5%Mo-CN, and (**e**, **f**) 15%Mo-CN nanotubes.

FE-SEM studies the surface morphology of pristine g-C3N4, 5%Mo-CN, and 15%Mo-CN nanotubes, and the respective scanning electron micrographs are shown in Fig. 2. Both pristine g-C_3_N_4_ and Mo-CN composite samples exhibit nanotubes-like surface morphology with variable length, thickness, and diameter. However, it is observed that the pristine g-C_3_N_4_ nanotubes exhibit the finest surface morphology compared to Mo-CN samples. For example, the average diameter of g-C_3_N_4_ nanotubes is 154 ± 28 nm, while their length varies from 500 nm to a few µm, as shown in Fig. 2a, b. Furthermore, the impregnation of Mo nanoparticles on g-C_3_N_4_ nanotubes in a hydrothermal process (180 °C for 24 h) also leads to the thickening and growth of g-C_3_N_4_ nanotubes. For instance, the average diameter of 5%Mo-CN nanotubes increased to 196 ± 43 nm. However, this effect is more pronounced at the higher concentration of ammonium molybdate heptahydrate, the precursor used for the fabrication of Mo nanoparticles, because 15%Mo-CN nanotubes exhibit a significant increase in the thickness, length, and diameter compared to 5%Mo-CN or pristine g-C_3_N_4_ samples. As can be seen in Fig. 2e, f, 15%Mo-CN nanotubes are 5 µm long with relatively smooth walls, and their diameter is 500 ± 75 nm. Thus, ammonium molybdate heptahydrate concentration significantly influences the morphology of Mo-CN nanotubes.

**Figure 3:**
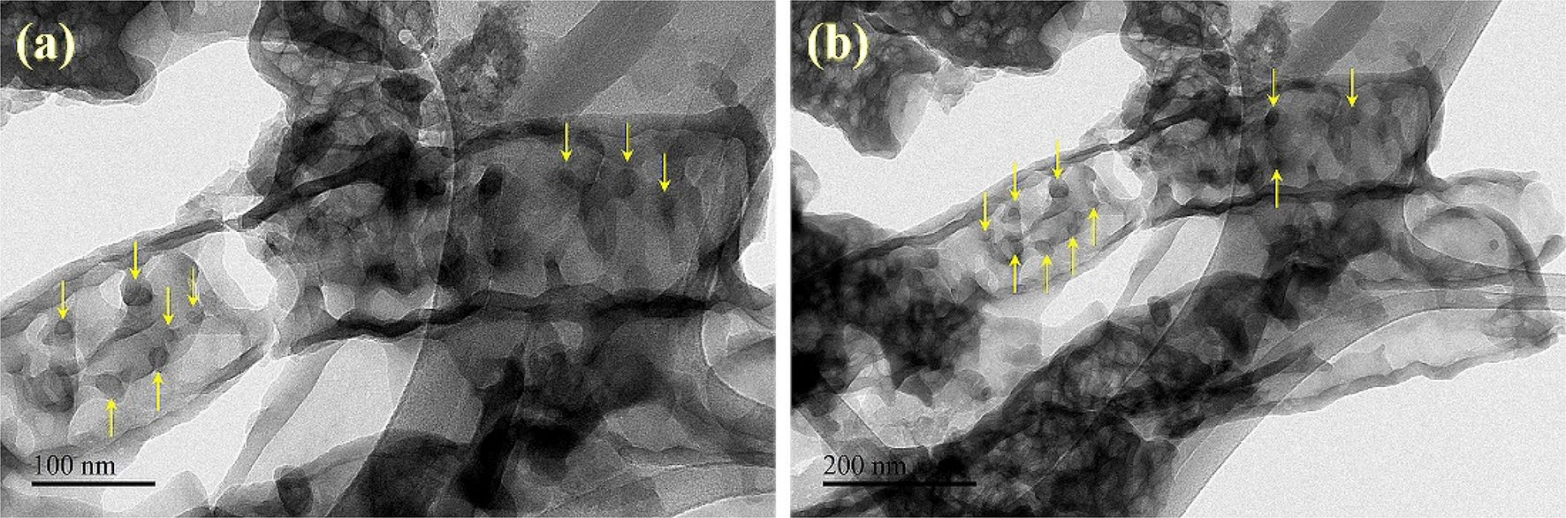
HR-TEM images of 5%Mo-CN sample show Mo nanoparticles' impregnation on g-C_3_N_4_ nanotubes (**a**) HR-TEM micrograph at 100 nm (**b**). HR-TEM micrograph at 200 nm.

The structure and impregnation of Mo into g-C_3_N_4_ nanotubes are further revealed by transmission electron microscopy. Figure 3a, b shows the HR-TEM images of 5%Mo-CN nanotubes. Under high magnification, Mo nanoparticles incorporated into the planes/coordinates of the g-C_3_N_4_ nanotubes can be observed. The dimensions of Mo nanoparticles embedded into Mo-CN nanotubes is around 10 nm. The HR-TEM micrographs at 100 nm and 200 nm expose the uniform distribution of Mo in the obtained Mo-CN nanotubes. The elemental presence of metallic Mo, Mo(IV), C, and N is further supported by XPS analysis (Discussed in detail in Sect. 3.4 and Fig. 5). The Mo3d core-level spectrum indicates two doublets because of the low spin–orbit splitting. These doublets are distinguished as metallic Mo and Mo(IV) as oxide or hydroxide with peaks at respective binding energies. These findings emphasize the impregnation of Mo into g-C_3_N_4_ nanotubes and reveal the presence of both metallic and Mo (IV) in Mo-CN nanotubes.

### X-ray diffraction

**Figure 4:**
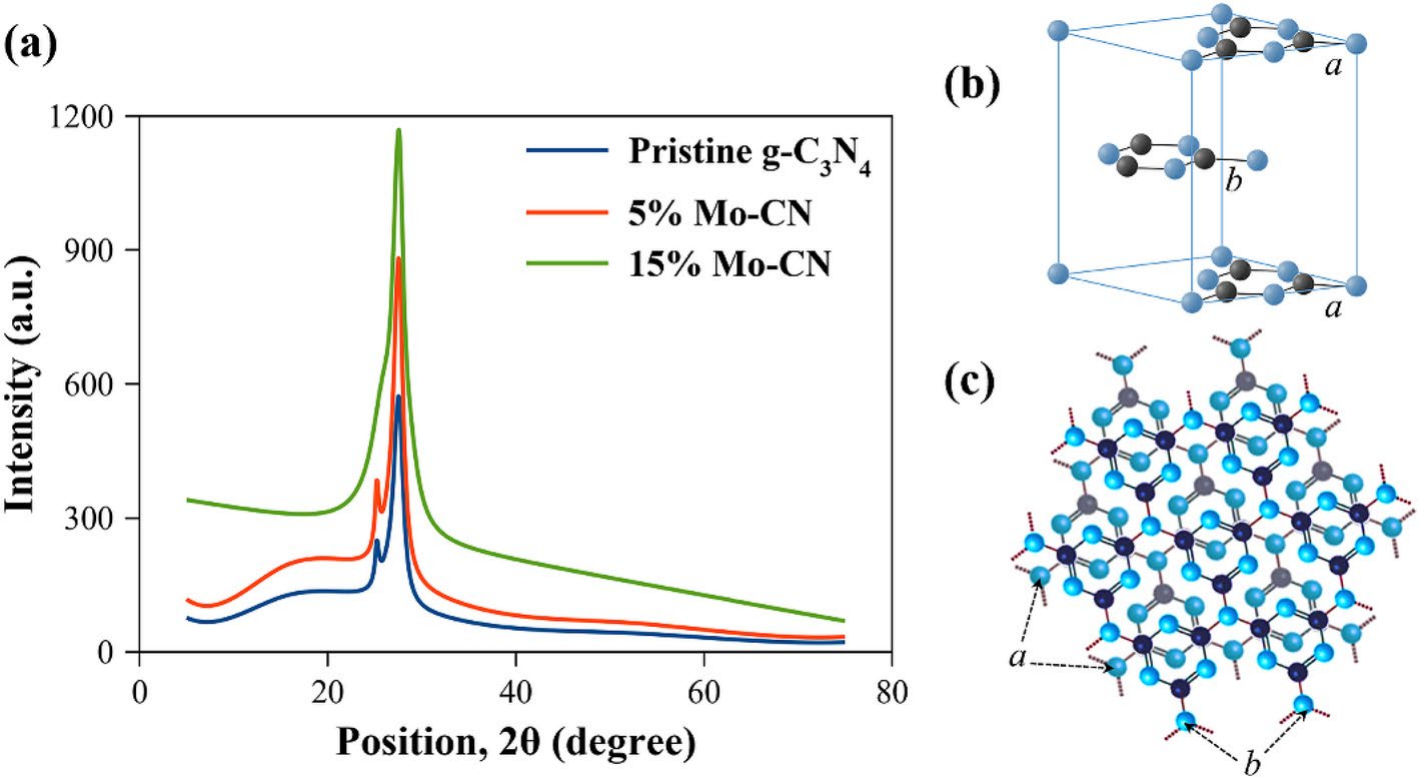
(**a**) XRD patterns of the as-prepared, pristine g-C_3_N_4_ and Molybdenum impregnated g-C_3_N_4_ samples. (**b**) The unit cell, formed by AB-stacking of planar graphitic layers shown in (**c**).

XRD investigates the structural purity and crystalline phase analysis of pristine g-C_3_N_4_ and Mo-CN nanotubes. Figure 4 shows the XRD patterns of the pristine g-C_3_N_4_, 5%Mo-CN, and 15%Mo-CN nanotubes. The characteristic sharp peak at 27.5° and a hump at 13° 2θ (Cu Kα radiation) corresponding to the (002) and (100) are the reflections of crystalline g-C_3_N_4_ layered material that further reveals the formation of triazine-based g-C_3_N_4_ structure. That is in good agreement with the ICDD reference database for g-C_3_N_4_, JCPDS 87–1526^21^. Moreover, Suter et al.^53^ estimated the XRD patterns of planar-/buckled-layer configurations of triazine-based g-C_3_N_4_ from DFT calculations and concluded that the planar graphitic layers with AB-stacking and interlayer spacing of ~ 3.24 Å exhibit the main peak around ~ 27° 2θ with a minor sharp reflection at 24.4° 2θ. We observed similar patterns for pristine g-C_3_N_4_ and 5%Mo-CN nanotubes with reflections at 27.5° and 25.2° 2θ and an interlayer spacing of 3.24 Å. In the case of 15%Mo-CN nanotubes, only the main reflection at 27.5° 2θ is observed with no shoulder around ~ 24.4° 2θ, which may be attributed to the modification of the layered structure during the hydrothermal impregnation of Mo nanoparticles. In addition, the degree of crystallization of Mo-doped g-C_3_N_4_ catalysts reduces apparently with increasing Mo concentration, also illustrated by Wang et al.^21^ for fabrication of Mo dopped g-C_3_N_4_. Nonetheless, our results are consistent with the x-ray data of AB-stacked triazine-based g-C_3_N_4_ with the space group P6̅m2 and conform with the relevant experimental and computational studies. Furthermore, the XRD patterns do not reflect Molybdenum or its compounds, such as molybdenum oxides or molybdenum nitrides, indicating that the Mo species are embedded into in-planes and coordinate to the g-C_3_N_4_ matrix by Mo–N bonds^23,54^. Since the concentration of impregnated Mo nanoparticles is low, they are not detected due to the sensitivity limitations of powder XRD. For this purpose, X-ray photoelectron spectroscopy (XPS) is performed, and the results are discussed in Sect. 3.4.

### X-ray photoelectron spectroscopy

**Figure 5:**
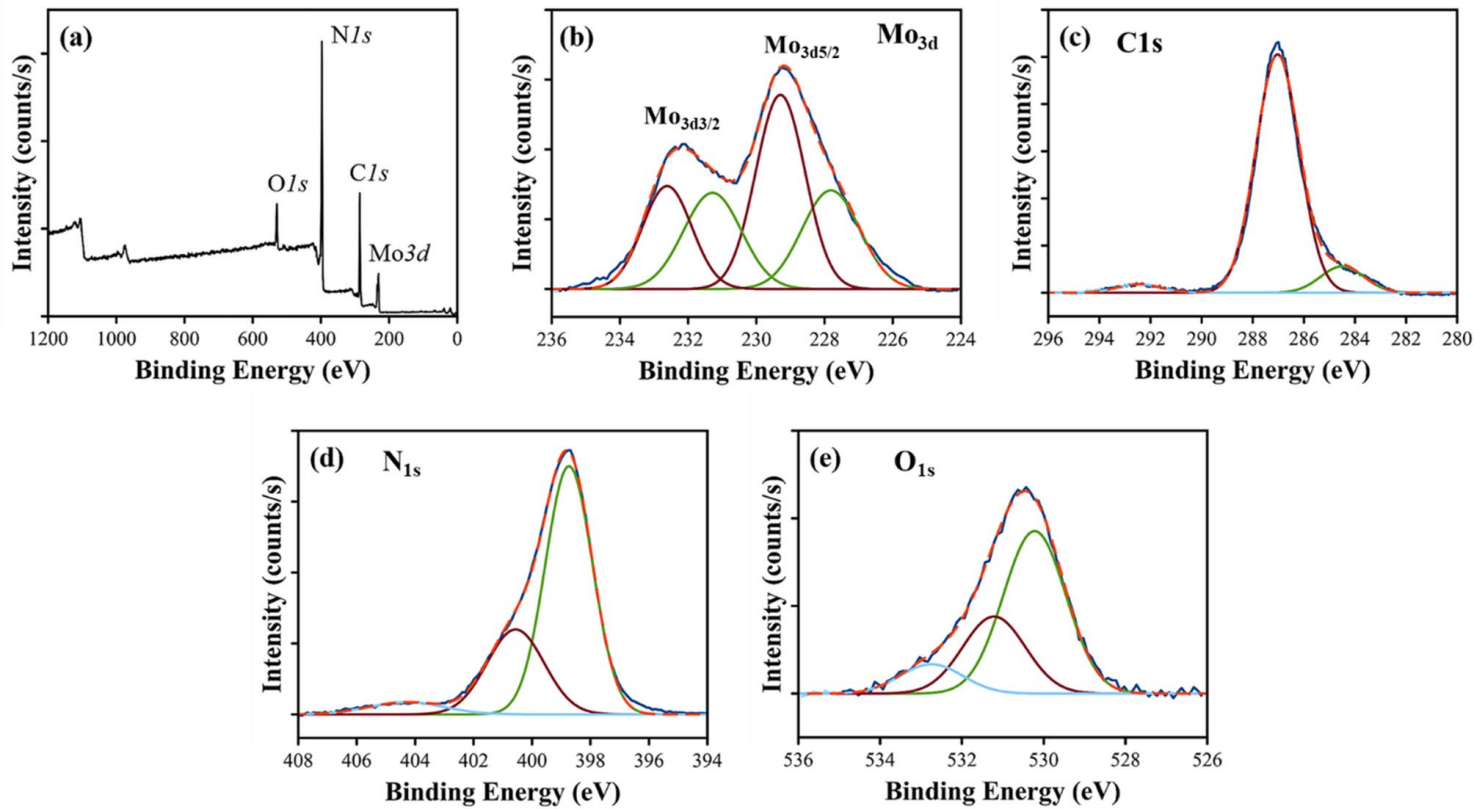
X-ray photoelectron spectroscopy: (**a**) XPS survey scan of 5%Mo-CN nanotubes; and (b-e) the core-level XPS spectra of (**b**) Mo3d, (**c**) C1s, (**d**) N1s, and (**e**) O1s showing the chemical state and speciation of different elements.

XPS is used to confirm the formation of Mo-CN composite nanotubes and elemental states. Figure 5 shows the XPS survey scan and the characteristic core-level spectra of Mo3d, C1s, N1s, and O1s as a function of the respective binding energy values, which agree with the relevant literature^21,37,55^. The C1s peak at 284.5 ± 0.1 eV is used as a charge reference for the XPS core-level spectra and is usually attributed to the carbon-containing contaminations or adventitious carbons^56^. In the C1s core-level spectrum, as shown in Fig. 5c, a major peak observed at 287.0 ± 0.1 eV (FWHM = 2.0 eV) is the characteristic of the sp^2^-hybridized carbon atoms. The π-excitation also confirms the presence of C=N double bonds at 292.5 ± 0.1 eV^56^. The deconvolution of the Mo3d core-level spectrum indicates two doublets because of the low spin–orbit splitting (~ 3.3 ± 0.1 eV) among Mo_3d_ core excitations (3/2 and 5/2), as shown in Fig. 5b. These doublets are distinguished as the metallic Mo with peaks at 227.9 ± 0.1 eV for Mo3d_5/2_ and 231.2 ± 0.1 eV for Mo3d_3/2_ (FWHM = 2.0 eV) and Mo(IV) as oxide or hydroxide with peaks at 229.3 ± 0.1 eV for Mo3d_5/2_ and 232.6 ± 0.1 eV for Mo3d_3/2_ (FWHM = 1.7 eV)^36,57^.

Three components are identified in the N1s core-level spectrum, as shown in Fig. 5d. These components are distinguished as: (a) a peak at 398.8 ± 0.1 eV (FWHM = 1.9 eV) corresponding to the sp^2^-hybridized aromatic C=N–C, (b) a peak at 400.6 ± 0.1 eV (FWHM = 2.3 eV) attributed to the ternary C–N(–C)–C (fully condensed) units, and (c) a weak peak at 404.3 ± 0.1 eV (FWHM = 3.0 eV) corresponding to the C–NH–C (partially condensed) units^58^. The O1s core-level spectrum reveals three components, as shown in Fig. 5e, which are identified as metal (Mo(IV)) oxides with a characteristic peak at at 530.2 ± 0.1 eV (FWHM = 1.8 eV), metal (Mo(IV)) hydroxides showing a peak at 531.2 ± 0.1 eV (FWHM = 1.8 eV), and adsorbed water appearing at 532.7 ± 0.1 eV (FWHM = 1.8 eV)^59,60^. These results not only confirm the impregnation of Mo nanoparticles on g-C_3_N_4_ nanotubes and the formation of Mo-CN composites but reveal the presence of both metallic and oxidic Mo nanoparticles. Chemical speciation of the Mo3d core-level spectrum demonstrates a ratio of 1:2 between the metallic Mo nanoparticles and Mo(IV) oxide/hydroxide species. On the other hand, the characteristic triazine-based structure of g-C_3_N_4_ nanotubes is confirmed from the core-level C1s and N1s spectra, which align well the previously published reports^36,38,49,56–58,61^.

### UV–visible spectroscopy

**Figure 6:**
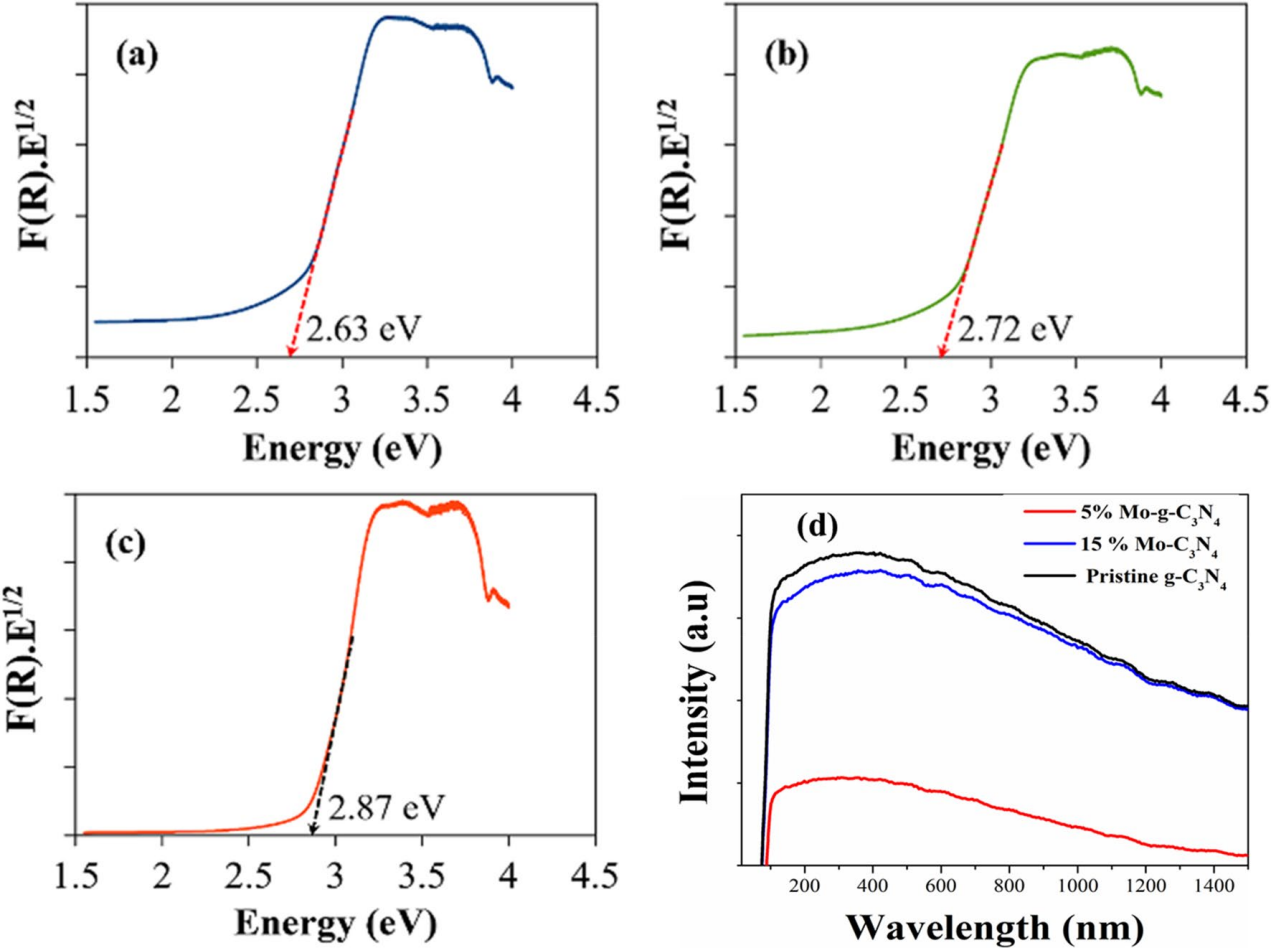
The bandgap energy estimation from UV–vis diffused reflectance spectroscopy of the as-prepared pristine g-C_3_N_4,_ and Molybdenum impregnated g-C_3_N_4_ samples: (**a**) 5%Mo-CN, (**b**) pristine g-C_3_N_4_, and (**c**) 15%Mo-CN nanotubes. (**d**) The PL spectra of Pristine g-C_3_N_4_, 5% Mo-g-C3N4, and 15% Mo-g-C3N4, respectively.

UV–visible spectroscopy is performed to study the optical properties of pristine g-C_3_N_4_ and Mo-CN composite nanotubes. Figure 6 shows the UV–vis diffused reflectance spectra of different samples. To calculate the bandgap, we have used Kubelka–Munk (K–M) function and Tauc plots^62–64^. The K-M function and reflectance F(R) were used to calculate (*R*).*E*^1/2^ factor. In order to find out bandgap energy, (*R*).*E*^1/2^ was plotted against the photon energy (eV). For indirect energy transitions (*F*(*R*).*E*^1/2^), the bandgap energy was calculated from the point where the energy coordinate on the lower energy side of the F(R) curve showed a linear increase. Extrapolating a straight line from the F(R) curve towards the x-axis gives the value of the bandgap, as shown in Fig. 6a–c. The bandgap energy of 5%Mo-CN, pristine g-C_3_N_4_, and 15%Mo-CN samples is estimated to be 2.63, 2.72, and 2.87 eV, respectively.

### Photoluminescence spectroscopy

The room temperature photoluminescence spectra for the Mo Impregnated g-C_3_N_4_ are recorded at an excitation wavelength of 350 nm, as shown in Fig. 6d. The outstanding photocatalytic performance of the 5%Mo-impregnated catalysts can be attributed to its nanotubes-like structure and narrow bandgap, which allows it to harvest light more efficiently. In Mo-CN nanotubes, Molybdenum may act as the photogenerated electron target to reduce the recombination of photogenerated electron–hole pairs. This observation and the separation efficiency of the photogenerated electrons and holes are confirmed by photoluminescence is also supported by impedance analysis (Fig. 8). The maxima of the PL peak are observed around ~ 450 nm, which lies in the visible light region.

On the other hand, the pristine g-C_3_N_4_ exhibits a strong emission peak at about ~ 450 nm at ambient temperature. It is a known fact that higher fluorescence intensity means more recombination of electron–hole pairs and lower photocatalytic activities^65^. The carrier dynamics of pristine g-C_3_N_4_ and 5%Mo-CN and 15% Mo-CN are presented in Fig. 6d. In contrast, all the samples exhibited similar emission peaks in the range of − 450 nm. The emission intensities of 5% Mo-CN and 15% Mo- CN were lower than that of pristine g-C_3_N_4_^21,66^. This is indicating the enhanced charge separation, and transfer in Mo impregnated CN nanotubes. The lowest peak intensity of 5% Mo-CN indicated suppressed charge recombination, highest charge separation, and transfer efficiency, thereby definitely preferred the photocatalytic water splitting process.

### Photoelectrochemical (PEC) measurements

**Figure 7:**
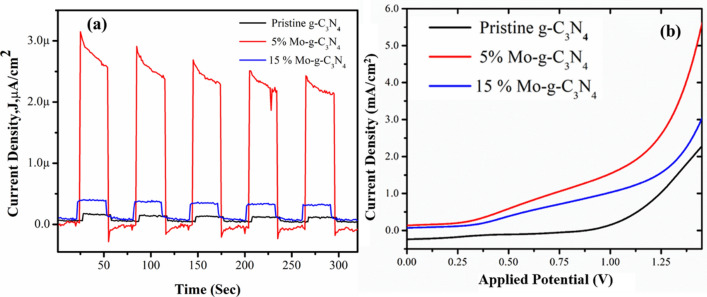
(**a**) The photocurrent profiles recorded for the as-prepared, pristine g-C_3_N_4_ and Molybdenum impregnated g-C_3_N_4_ samples: pristine g-C_3_N_4_, 5%Mo-CN, and 15%Mo-CN nanotubes. (**b**) The current density vs. applied potential profiles, recorded under 1 SUN irradiation for the pristine g-C_3_N_4_ and Molybdenum impregnated g-C_3_N_4_ samples using linear sweep voltammetry.

FTO photoanodes coated with pristine g-C_3_N_4_ and Mo-CN composite nanotubes are used for PEC measurements in a standard three-electrode system consisting of a reference (SCE) electrode, counter (Pt wire) electrode, and working (FTO) electrodes. 0.5 M Na_2_SO_4_ at neutral pH (7) is used as the electrolyte solution. The cell is exposed to a solar irradiation source (1 SUN with AM filter 1.5G) at regular intervals to record photoresponse. Based on the initial cyclic voltammetry experiments, chronoamperometry is performed to study the photoresponse of pristine g-C_3_N_4_ and Mo-CN composite nanotubes. Figure 7a shows the photocurrent density vs. time (J*p* vs. t) profiles of these nanomaterials. At the same scale, the photoresponse of 5%Mo-CN/FTO electrode is the highest, i.e., ~ 3 µA at an applied potential of 0 V. Consequently, 5%Mo-CN nanotubes exhibit ≥ 5 times higher photocurrent compared to pristine g-C_3_N_4_ and 15%Mo-CN nanotubes at 0 V. Furthermore, these nanostructures exhibit reversible photocurrent with excellent stability under on/off visible light illuminations. This, in turn, reflects the successful performance of 5%Mo-CN nanotubes composite material for effectual water splitting processes.

The linear sweep voltammetry experiments further complement this study, where the photocurrent density enhances to ~ 5.5 mA at higher potential values recorded for 5%Mo-CN nanotubes. Figure 7b shows comparative linear sweep voltammetry profiles of pristine g-C_3_N_4_ and Mo-CN composite nanotubes coated on FTO substrates. The measurements are performed in the potential range of 0–1.5 V against standard Ag/AgCl reference electrode under simulated visible light (1 SUN) illumination at a scan rate of 100 mV/s. Figure 7b shows that the current density of 5%Mo-CN nanotubes increases with applied potential and is estimated to be 5.5 mA/cm^2^. Compared to 15%Mo-CN and pristine g-C_3_N_4_ nanotubes, and it is 2–3 times higher. It is believed that the higher the current density, the higher the electron/hole (e^−^/h^+^) separability, which in turn improves the photocatalytic activity. Therefore, the transient photocurrent measurements and linear sweep voltammetry also substantiate the charge transfer dynamics at the g-C_3_N_4_ nanotubes and Mo nanoparticles interface.

Consequently, the highest photoresponse and current density of 5%Mo-CN nanotubes at various potentials can be ascribed to the lower bandgap, high surface area, and superior interfacial charge transfer dynamics compared to 15%Mo-CN nanotubes. Moreover, considering the positive potential response, the oxygen evolution reaction (OER) is the preferred photoelectrochemical process observed during the linear sweep voltammetry measurements. Hence, 5%Mo-CN nanotubes demonstrate high photocurrent efficiency and photochemical oxygen evolution performance that is the most impressive achievement acclaimed in PEC studies.

**Figure 8:**
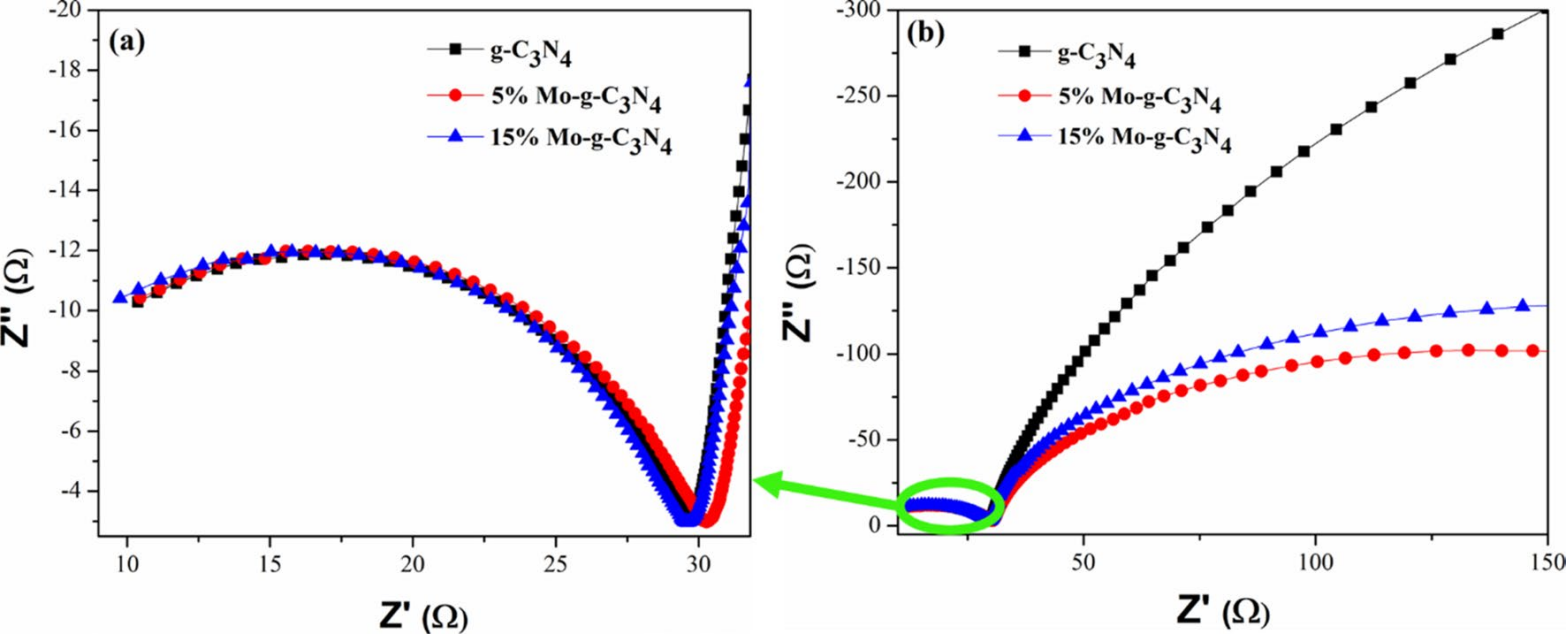
EIS studies of as prepared g-C_3_N_4_ Nanotubes and Mo-Impregnated g-C_3_N_4_ Nanotubes Photocatalyst (**a**) Magnified view at the high frequency of 5% Mo- g-C_3_N_4_ nanotubes presenting lowest charge transfer resistance (**b**) The semicircle of % Mo- g-C_3_N_4_ is at a lower position in the Nyquist plot as compared to 15% Mo- g-C_3_N_4_ and g-C_3_N_4_ at low frequency.

The electrochemical impedance spectroscopy (EIS) was performed to investigate the interfacial charge transfer kinetics, such as the efficiency of blocking the recombination of photoinduced electron and hole pairs by the pristine g-C_3_N_4_ nanotubes and Mo- impregnated g-C_3_N_4_ nanotubes respectively. Figure 8a, b shows the EIS spectra of pristine g-C_3_N_4_ and 5% Mo- g-C_3_N_4_ and 15% Mo- g-C_3_N_4_ samples at higher frequency Fig. 8a and low frequencies Fig. 8b. Several studies ^67–69^ illustrated that the semicircle diameter in a Nyquist plot is proportional to the charge-transfer resistance of the material under observation, providing valuable information on charge transfer processes. Hence, the smallest semicircle is observed for 5% Mo- g-C_3_N_4_ in the Nyquist plot compared to 15% Mo- g-C_3_N_4_ and g-C_3_N_4_, as shown in Fig. 8b. Thus, illustrating the low resistance for charge transport for 5% Mo- g-C_3_N_4_. On the other hand, the semi-circular Nyquist plots showed the largest diameter for g-C_3_N_4_ and then for 15% Mo- g-C_3_N_4_. Therefore, we can conclude that 5% Mo- g-C_3_N_4_ nanotubes possess the lowest charge transfer resistance (R_*ct*_) among g-C_3_N_4_ and 15% Mo-g-C_3_N_4_, which further confirms the fast electron transfer kinetics and thus efficient separation of photogenerated e − -h + pairs. Additionally, the small arc radius also demonstrates the fast interfacial charge transfer efficiency for 5% Mo- g-C_3_N_4_. Thus, the EIS observations are in line with the enhanced photocatalytic activity of the 5% Mo- g-C_3_N_4_ Nanotubes compared to its counterparts (Fig. 7a, b). This is credited to photogenerated carriers in the Mo-dopped materials with higher separation efficiency than the pristine g-C_3_N_4_ nanotubes and 15% MO- g-C_3_N_4_ nanotubes. It is important to mention here, sometimes by increasing the amount of a doped metal from a certain level, the photocatalytic recital could not be improved. Guo et al. demonstrated this phenomenon; with higher doping amounts of metal, new recombination centers for carriers electrons and holes are created, limiting the photocatalytic performance of the photocatalyst^39^. Therefore, the lower performance of g-C_3_N_4_ nanotubes is certainly due to the absence of Mo doped metal, but for 15% Mo- g-C_3_N_4_ nanotubes, the decreased photoactivity could be due to higher Mo content.

### Proposed charge-transfer mechanism

PEC studies reveal that Mo-impregnated g-C_3_N_4_ nanotubes possess excellent structural and electronic properties that fulfill the prerequisites for an efficient heterogeneous photoelectrocatalyst. The 5%Mo-CN nanotubes exhibit precise nanostructure/morphology (Figs. 2 and 3) and electronic properties with an appropriate bandgap of 2.63 eV, calculated from the Kubelka–Munk (K-M) function and Tauc plots (Fig. 6). Its bandgap is satisfactorily high to overcome the endothermic atmosphere of the water-splitting reaction. It is commonly recognized that theoretically estimated energy must be higher than the endothermic character of the water-splitting reaction to produce H_2_ from water. This is further explained in Eqs. (1) and (2), as given below:

1 $$2{\text{H}}_{2} {\text{O}} \to {\text{O}}_{2} + 4{\text{H}}^{ + } + 4{\text{e}}^{ - } + 0.82 {\text{V}}$$

2 $$2{\text{H}}^{ + } + 2{\text{e}}^{ - } \to {\text{H}}_{2} - 0.41\,{\text{V}}$$

From these equations, it is clear that a photocatalyst requires a minimal amount of energy equivalent to 1.23 V of redox potential to initiate the water-splitting reaction, i.e., H_2_ production. Hence, to start solar-driven water splitting with a photoelectrocatalyst, the material's bandgap must be larger than this minimal energy. Sometimes, the intermediates formed during the 4-electron transfer reaction have higher energy; therefore, a certain inherent overpotential must be well-thought-out. This makes photons exceeding about 1.8 eV energy or higher that is practically suitable for water splitting^21,25,37,70^. Light absorption in photoelectrochemical reactions is associated with various instant molecular relaxations taking place between the material and the solvent thus, the bandgap of the photocatalyst should be in such a range that a photogenerated electron must have sufficient reduction power to reduce water to H_2_ and the photogenerated hole has enough oxidation strength to oxidize water to O_2_^21,37,70^.

Figure 2S (Supplementary information) shows the proposed mechanism of electron/hole (e^−^/h^+^) transfer in the 5%Mo-CN nanotubes. It is believed that the observed bandgap energy of 2.63 eV carries both the half-cell reactions independently. This type of mechanism is rarely observed in organic semiconducting material and indicates that 5%Mo-CN nanotubes are stable in water and can perform efficient visible-light-driven water splitting. This is because of the suitable microstructure of 5%Mo-CN nanotubes, smaller size and thereby, larger surface area (compared to 15%Mo-CN nanotubes), and better interfacial charge transfer dynamics. Also, the presence of a high concentration of sp^2^-hybridized nitrogen atoms, responsible for electron localization, ultimately shows high photocurrent efficiency. Furthermore, the impregnation of Mo nanoparticles on the g-C_3_N_4_ surface also enhances the visible light-harvesting ability of g-C_3_N_4_ nanotubes materials and optimized charge separation, which is also attributed to the reduction in bandgap energy (Eg) level^39^.

The crystalline nature of 5%Mo-CN nanotubes is also believed to improve the PEC properties by promoting the kinetics of charge diffusion in both the bulk and on the surface^11,71^. It is generally observed that g-C_3_N_4_ is an effective photocatalyst for water splitting under visible light, but its efficiency can be further enhanced by introducing some sacrificial electron donor/acceptor or by doping it with transition metal or noble metal catalysts^11,71^. In this study, the impregnation of Mo nanoparticles on g-C_3_N_4_ nanotubes also enhanced the photocurrent efficiency in a similar way for water reduction into H_2_ or water oxidation into O_2_. It is observed that the oxygen evolution reaction (OER) is dominant in this case in the positive potential range due to kinetic effects^11,38,71,72^. Hence 5% Mo-CN nanotubes generated better photocurrent under 1 SUN visible light irradiation.

## Conclusions

This article reports a facile strategy of synthesizing Mo impregnated g-C_3_N_4_ nanotubes from melamine and illustrated its characterization by different spectroscopic, microscopic, and electrochemical techniques. As a result, 5%Mo-CN nanotubes exhibit low bandgap (2.63 eV), high nitrogen concentration, and excellent photoelectrochemical properties compared to pristine g-C_3_N_4_ and 15%Mo-CN. Furthermore, the XRD pattern of 5%Mo-CN nanotubes revealed the crystalline structure of triazine-based g-C_3_N_4_ that remained intact during the hydrothermal process. Simultaneously, XPS discloses the impregnation of Mo nanoparticles on g-C_3_N_4_ nanotubes and the presence of metallic Mo, Mo(IV), C, and N in 5%Mo-CN nanotubes. These characteristics of 5%Mo-CN nanotubes ultimately helped in achieving better optical and photoelectrochemical properties. Furthermore, the 5%Mo-CN nanotubes exhibit a high current density of 5.5 mA/cm^2^ and stable, repeatable photoresponse under visible-light illumination. The improved photocatalytic recital of 5% Mo-CN could be explained by its structural feature and carrier kinetic properties. When irradiated under visible light, increased light absorption can be achieved, and this is credited to the narrowed bandgap energy by Mo doping in g-C_3_N_4_ nanotubes. Hence, more photogenerated carriers can easily jump in the conduction band due to decreased bandgap energy. Furthermore, enhanced charge carrier kinetics also improved the charge transfer and separation ability and made abundant electrons for redox reactions. The nanotube-like morphology of Mo impregnated g-C_3_N_4_ enlarged the surface area, providing more surface active sites and channels for photocatalytic activities. Henceforth, 5%Mo dopped g-C_3_N_4_ nanotubes composite is a potential photocatalyst for renewable energy applications.

## Supplementary Information

Supplementary Information.
