# Supplementary material for: Molybdenum impregnated g-C3N4 nanotubes as potentially active photocatalyst for renewable energy applications
Source: Sci Rep. 2021 Aug 19;11:16886. doi: 10.1038/s41598-021-96490-6 (PMC8377046; doi:10.1038/s41598-021-96490-6)
Supplement: Supplementary file 1 — Supplementary Information. [file 41598_2021_96490_MOESM1_ESM.docx]

**Supplementary Information**

Molybdenum impregnated g-C_3_N_4_ nanotubes as potentially active

photocatalyst for renewable energy applications

Naseer Iqbal ^1*^, Adeel Afzal ^1^, Ibrahim Khan ^2^, Muhammad Shahzeb Khan ^3^ and Ahsanulhaq Qurashi ^2,4^

1. Department of Chemistry, College of Science, University of Hafr Al Batin, P.O. Box 1803, Hafr Al Batin, 39524, Saudi Arabia;

[naseeriqbal@uhb.edu.sa](mailto:naseeriqbal@uhb.edu.sa); [aa@aafzal.com](mailto:aa@aafzal.com)

^2^ Center of Research Excellence in Nanotechnology, King Fahd University of Petroleum and Minerals, Dhahran, 31261, Saudi Arabia; [ibrahim.zarin@kfupm.edu.sa](mailto:ibrahim.zarin@kfupm.edu.sa)

^3^ Department of Mechanical Engineering, College of Engineering, University of Hafr Al Batin, P.O. Box 1803, Hafr Al Batin, 39524, Saudi Arabia; [mshahzeb@uhb.edu.sa](mailto:mshahzeb@uhb.edu.sa)

^4^ Department of Chemistry, Khalifa University of Science and Technology, Main Campus, Abu Dhabi, P.O. Box 127788, United Arab Emirates; [ahsan.qurashi@ku.ac.ae](mailto:ahsan.qurashi@ku.ac.ae)

* Correspondence: [naseeriqbal@uhb.edu.sa](mailto:naseeriqbal@uhb.edu.sa); Tel.: +966 (0) 13 720 3426.

**Additional Figures:**

The description and detailed discussion on figures S1 and S2 is provided in relevant sections of the main manuscript.





**Figure 1S.** FTIR spectra of the as-prepared, pristine g-C_3_N_4_ and molybdenum impregnated g-C_3_N_4_ samples (5%Mo-CN and 15%Mo-CN).


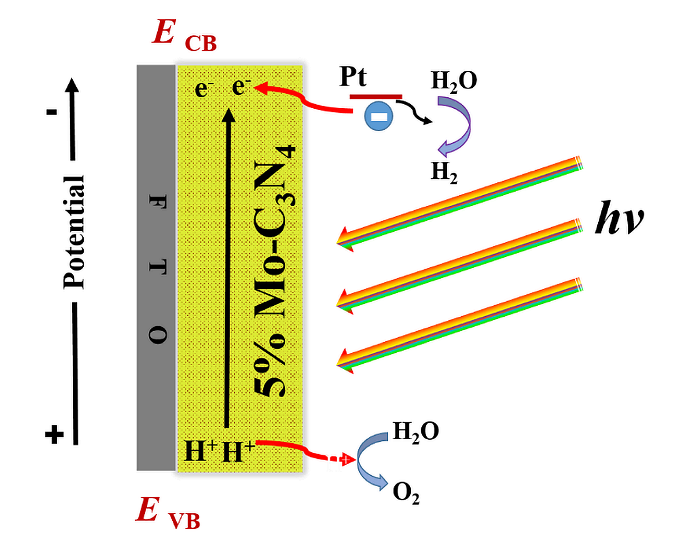


**Figure 2S.** Energy diagram of 5% Mo-CN nanotubes like photocatalyst (in 0.5 M Na_2_SO_4_ at pH 7) coated on FTO glass substrate showing plausible charge transfer mechanism upon exposure to 1 Sun (100mWcm−2AM 1.5G) irradiation source.
